# Spontaneous retrograde migration of a lower ureteric calculus in a child with primary non-refluxing obstructive megaureter: a rare case report

**DOI:** 10.1016/j.eucr.2026.103595

**Published:** 2026-09-07

**Authors:** Preet Jain, Piyush Singhania, Dhaval Desai, Swapnil V. Nikam

**Affiliations:** Department of Urology, MGM Medical College and Hospital, MGM Institute of Health Sciences, Mumbai, India

## Abstract

Primary non-refluxing megaureter associated with ureteric calculi in children is rare. Spontaneous retrograde migration of ureteric calculi is an exceptionally rare phenomenon, reported only in adults. 9-year-old boy presented with left flank pain and haematuria. Initial imaging demonstrated a 7-mm distal ureteric calculus with obstructive megaureter. Subsequent imaging unexpectedly revealed spontaneous retrograde migration of calculus to pelvis, without any history of stone expulsion in urine. Operative findings confirmed a narrow distal ureteric segment, consistent with primary non-refluxing obstructive megaureter. To our knowledge, this is the first reported paediatric case of spontaneous retrograde migration of a ureteric calculus in world literature.

## 1 Introduction

Primary megaureter is a congenital dilatation of the ureter arising from an intrinsic abnormality of the distal ureteric segment, in the absence of an anatomical bladder outlet obstruction or a neuropathic bladder. It is conventionally classified, on the basis of the presence or absence of reflux and obstruction, into refluxing, obstructed (non-refluxing), refluxing-and-obstructed, and non-refluxing non-obstructed subtypes. Primary non-refluxing obstructive megaureter, in which an aperistaltic narrow juxtavesical segment impairs urine transport while reflux is absent, accounts for 5-10% of antenatally detected hydronephrosis.

The coexistence of ureteric calculi with an obstructive megaureter is distinctly uncommon in children. When calculi do occur they are generally secondary to chronic urinary stasis within the dilated, poorly draining ureter, they frequently remain asymptomatic until adolescence or adulthood. Under normal physiological conditions, coordinated antegrade ureteric peristalsis propels both urine and any calculus from the kidney towards the bladder. Movement of a calculus in the opposite, retrograde direction—from the ureter back towards the kidney—runs counter to this physiology and is therefore exceptionally rare; the few cases described in the literature have all occurred in adults.^1^, ^2^, ^3^, ^4^, ^5^, ^6^

We report what we believe to be the first case of spontaneous retrograde migration of a lower ureteric calculus in a child, occurring in association with a primary non-refluxing obstructive megaureter.

## 2 Case presentation

A 9-year-old boy presented to the urology outpatient department with left flank pain for 1 week. He also reported three episodes of gross, painless haematuria over the preceding one month. There was no history of fever, lower urinary tract symptoms, prior stone passage, or comparable illness in the past, and no relevant antenatal history or family history. General physical and abdominal examination was unremarkable, and routine haematological and biochemical investigations, including renal function, were within normal limits.

**Fig. 1:**
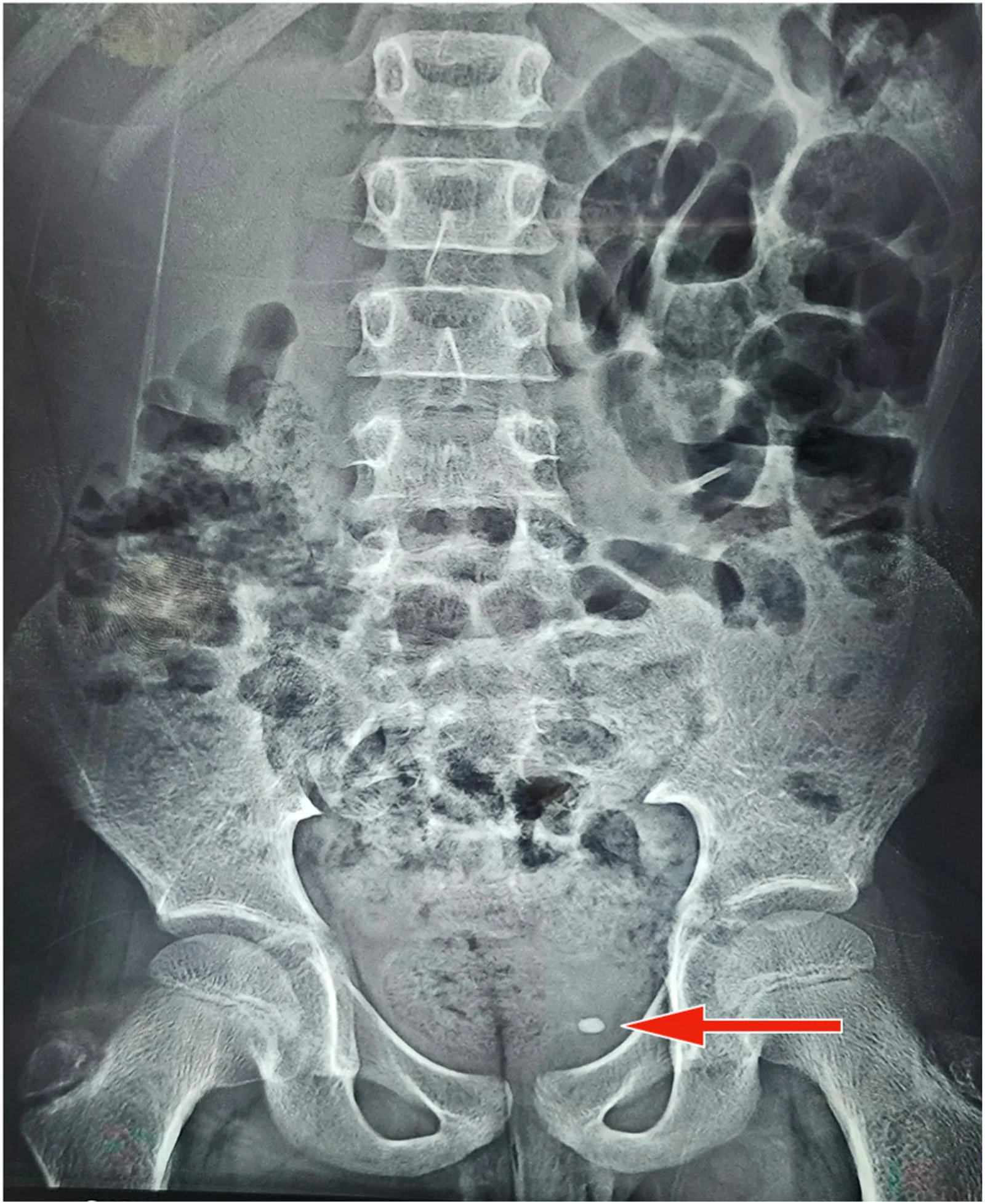
Initial plain KUB radiograph showing a solitary radio-opaque calculus projected over the left distal ureter (RED arrow).

Ultrasonography of the kidneys, ureters and bladder revealed a moderately hydronephrotic enlarged left kidney with thinning of the renal parenchyma, dilatation of the entire left ureter (hydroureter), and a 7 mm calculus in the left distal ureter. A plain radiograph of the kidneys, ureters and bladder (KUB) confirmed a solitary radio-opaque calculus projected over the left distal ureter (Fig. 1).

**Fig. 2:**
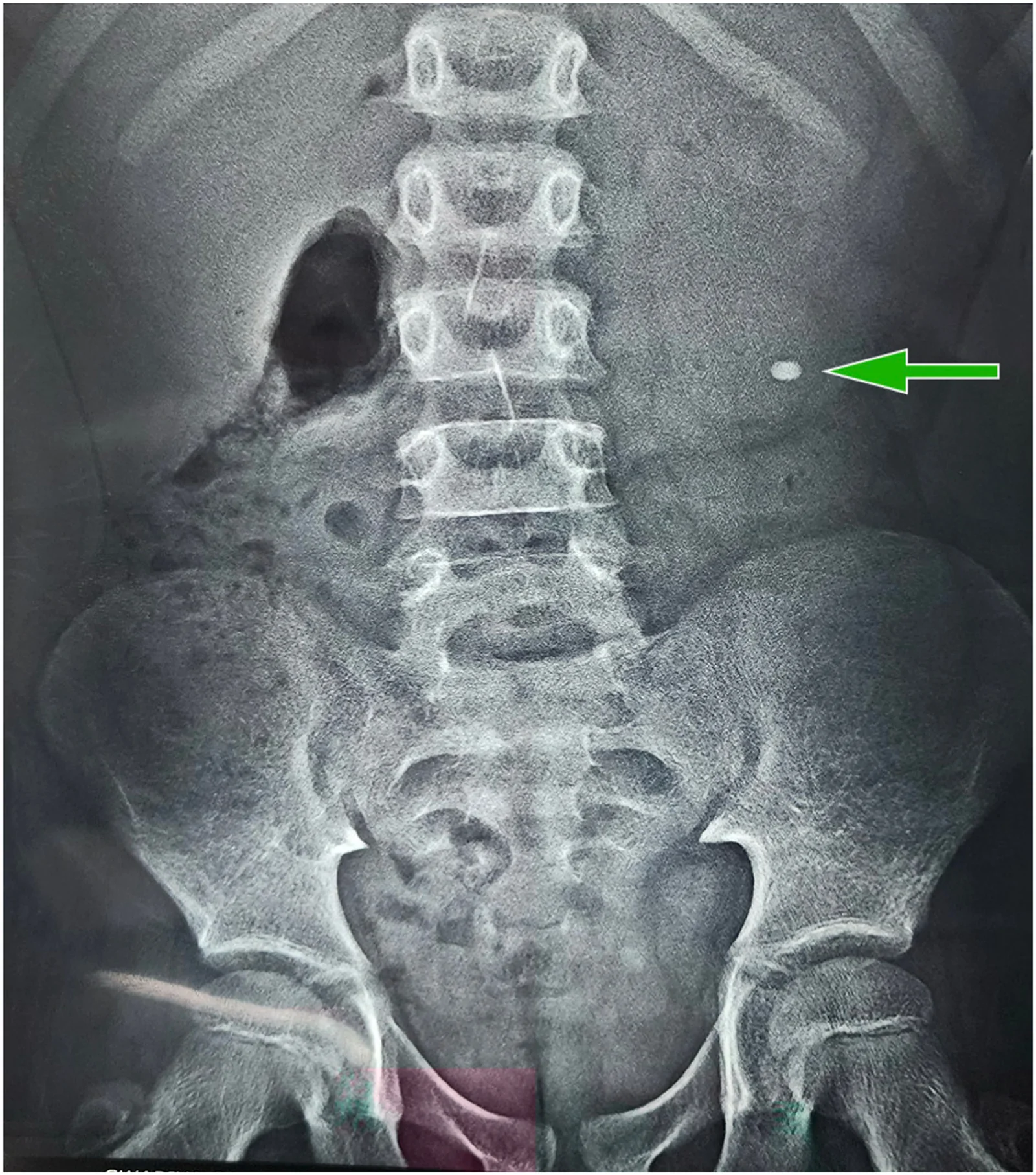
Subsequent radiograph/urogram showing the same calculus now located in the left renal region (GREEN arrow), confirming Spontaneous retrograde migration towards the kidney.

**Fig. 3:**
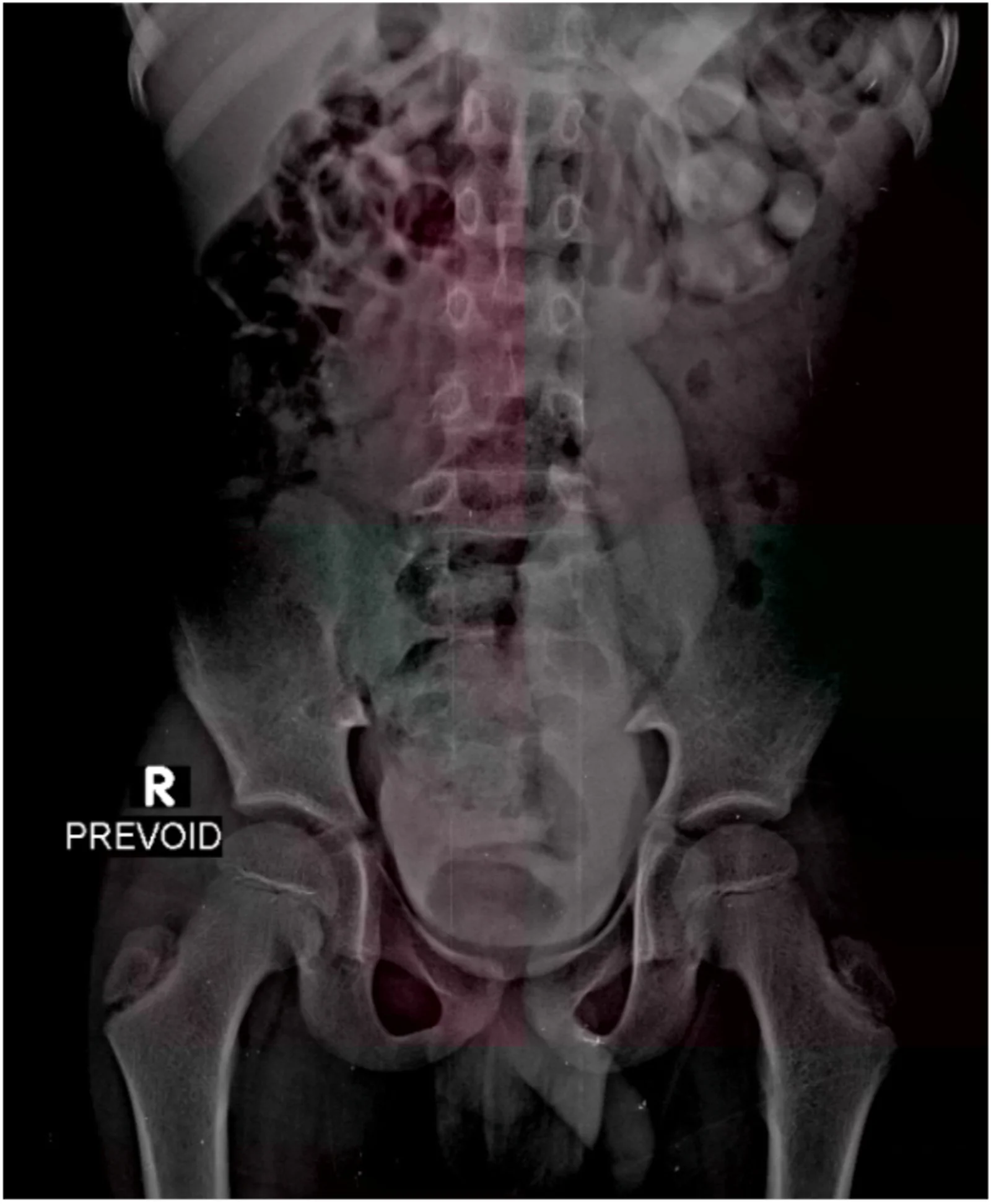
Prevoid film of Xray IVU demonstrating moderate left hydronephrosis with delayed contrast excretion.

Intravenous urography was performed to delineate the anatomy of the dilated system and functioning. Notably, on this study the calculus was identified in the renal region on scout image rather than in the distal ureter where it had been documented 12 days earlier on plain radiograph of the kidneys, ureters and bladder (Fig. 2) with no history of spontaneous passage of calculi in urine, indicating spontaneous retrograde migration of the calculus towards the kidney. It also demonstrated moderate left hydronephrosis with delayed contrast excretion (Fig. 3).

To characterise the underlying abnormality and to exclude associated pathology, further evaluation was undertaken. A micturating cystourethrogram was normal, with no vesicoureteral reflux and no posterior urethral valves. Technetium-99m diethylenetriamine pentaacetic acid (DTPA) diuretic renography demonstrated an obstructive drainage pattern on the left side with a preserved differential (split) function of 45%. The overall findings were consistent with a left primary non-refluxing obstructive megaureter with a secondary ureteric calculus.

**Fig. 4:**
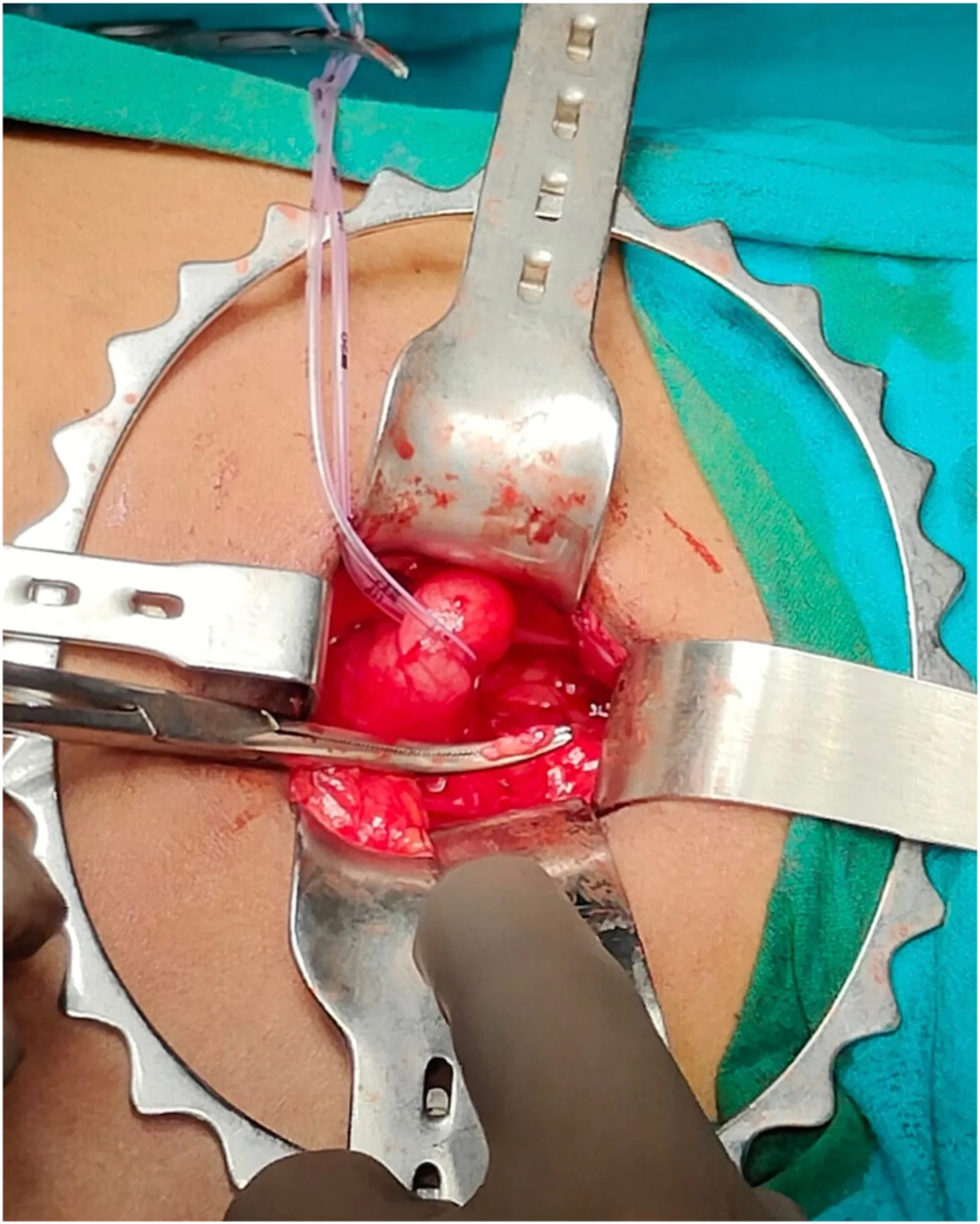
Intraoperative photograph showing a grossly dilated proximal ureter with a narrow distal segment near the Vesicoureteric junction. No calculus was palpable within the ureter.

In view of the obstructive non-refluxing megaureter, the child was scheduled for cystoscopy and open left ureteric exploration with Politano–Leadbetter reimplantation. During surgery, a grossly dilated proximal ureter with a narrow segment in the distal ureter close to the vesicoureteric junction was confirmed (Fig. 4); no calculus could be palpated along the course of the ureter, consistent with the preoperative imaging finding that the stone had migrated proximally into the kidney. Intraoperatively injection furosemide was administered as well but calculi could not be retrieved. Hence, a double-J stent was placed. The postoperative course was uneventful.

**Fig. 5:**
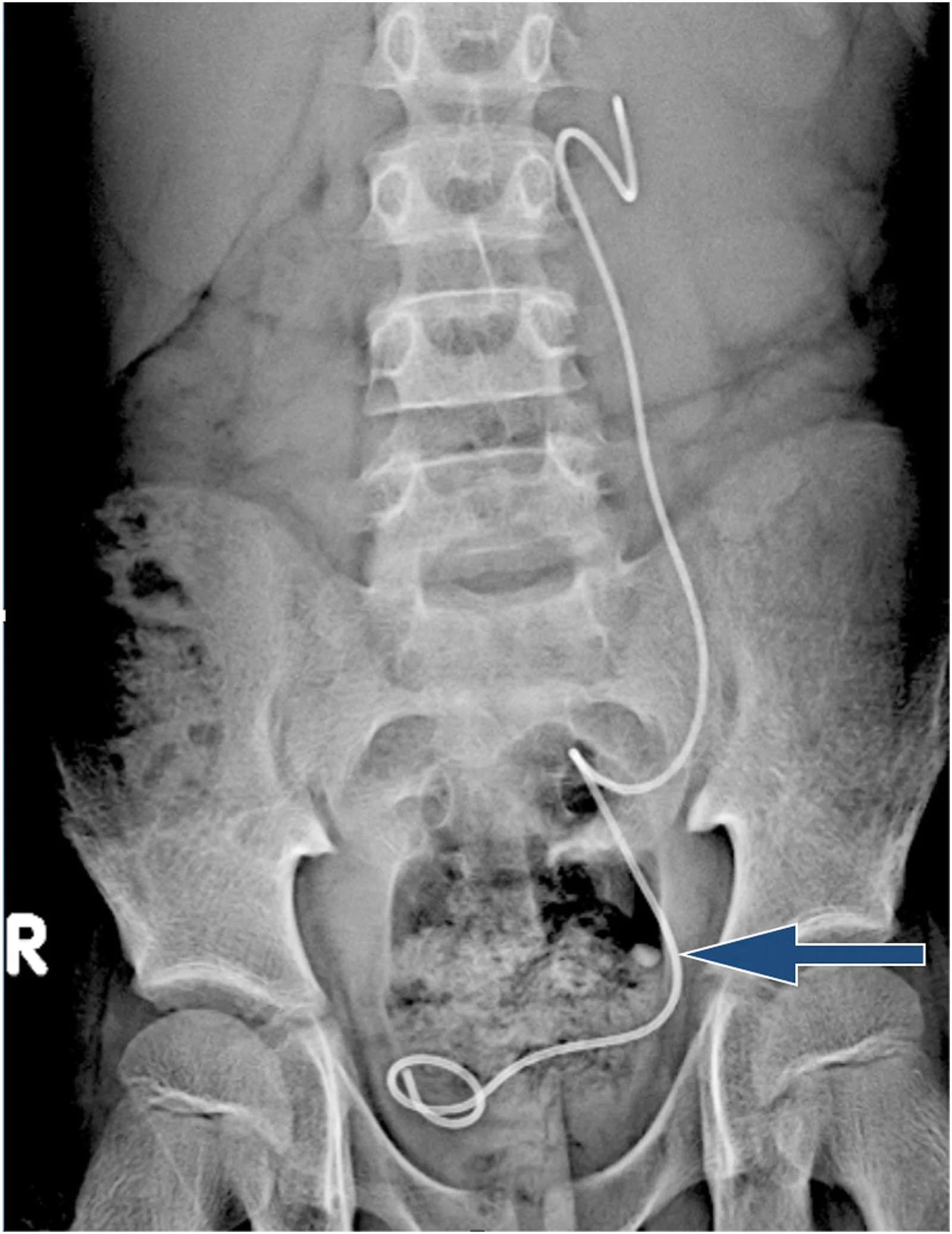
Plain KUB radiograph at six weeks showing the calculus (BLUE arrow) located in the left lower ureter once again, with the double-J stent in situ.

Six weeks later, a repeat plain KUB radiograph showed that the calculus had returned to the left lower ureter, with the double-J stent in situ (Fig. 5). The child was subsequently managed conservatively with oral Tamsulosin 0.2mg HS after which he spontaneously passed calculi in urine and then the stent was removed.

## 3 Discussion

Spontaneous retrograde migration of a ureteric calculus—movement of the stone proximally towards the kidney, against the direction of urine flow—is a rare and under-reported event. The handful of reports in the literature describe this phenomenon exclusively in adults. Patil et al. reported retrograde migration of a mid-ureteric calculus into the kidney as the first such case from India^2^; Khan et al. described a ureterovesical junction stone migrating to the kidney, reported as the first such case in the English literature^4^; Fallatah et al. documented upward migration of a ureteric stone in a military trainee^3^; Saliba et al. reported retrograde urolithiasis migration in a woman and proposed a possible mechanism^5^; and Miah et al. highlighted retrograde migration of a vesicoureteric junction calculus as a potential pitfall of limited non-contrast pelvic computed tomography.^6^ To our knowledge, no comparable case has previously been described in a child, nor in the specific setting of a primary non-refluxing obstructive megaureter.

The mechanism underlying retrograde stone migration is incompletely understood. It is hypothesised that uncoordinated or frankly reverse ureteric peristalsis, combined with reduced distal ureteric tone and altered intraluminal pressure dynamics, may permit a calculus to move proximally. The anatomy of an obstructive megaureter may be particularly conducive to this: the grossly dilated, poorly contractile proximal ureter and the narrow, functionally obstructing distal segment together create an environment of stasis and abnormal pressure gradients in which to-and-fro movement of urine—and of a small calculus suspended within it—can occur. In our patient, we postulate that a transient period of reverse ureteric peristalsis displaced the calculus from the distal ureter back towards the kidney. A similar phenomenon of retrograde urolith movement has been observed in canines and described in the veterinary literature, lending further plausibility to this explanation. The subsequent re-migration of the stone distally to the lower ureter by six weeks underscores the dynamic, bidirectional mobility of a calculus within such a dilated, dysfunctional system.

The clinical importance of recognising this phenomenon lies chiefly in avoiding diagnostic error. When a calculus that was previously documented in the distal ureter is no longer seen on repeat imaging, the change may be misinterpreted as spontaneous stone passage or complete resolution, potentially leading to inappropriate cessation of treatment or an inadequate operative plan. As this case demonstrates, the stone may instead have migrated retrograde and remain within the urinary tract. This emphasises the need for up-to-date imaging performed immediately before any planned intervention, careful correlation of serial studies, and vigilant follow-up, particularly in the anatomically abnormal megaureter where stone position can change unexpectedly.

The principal limitation of this report is that, as a single case, the proposed mechanism remains speculative and cannot be confirmed. Nonetheless, the case provides clear radiographic documentation of retrograde and subsequent antegrade calculus movement in a child with a primary non-refluxing obstructive megaureter.

## 4 Conclusions

To our knowledge, We report the first case of spontaneous retrograde migration of a lower ureteric calculus in a child, occurring within a primary non-refluxing obstructive megaureter. Clinicians should be aware that an apparent disappearance of a ureteric calculus from its previously documented position does not necessarily indicate stone passage; retrograde migration is a possibility, especially in dilated, obstructed systems. Repeat imaging immediately prior to surgery and meticulous follow-up are essential for accurate diagnosis and appropriate management.

## CRediT authorship contribution statement

**Preet Jain:** Writing – review & editing, Writing – original draft, Visualization, Resources, Methodology, Investigation, Formal analysis, Data curation, Conceptualization. **Piyush Singhania:** Project administration, Conceptualization. **Dhaval Desai:** Formal analysis, Data curation. **Swapnil V. Nikam:** Methodology, Formal analysis, Data curation.

## Consent to Participate and Ethics declaration

Written informed consent to take part in the study and to publish the article has been obtained from participant or his legal representative. The privacy right of participant has been observed.

This study was performed in compliance with relevant laws, regulatory frameworks and guidelines where the research took place. Ethics committee approval was not required under relevant laws and institutional guidelines. as per institutional protocol, no ethical committee approval is required for case report.The report was prepared in accordance with the Decalrations of Helsinki.

This research follows the CARE guidelines and the CARE checklist.

## Data availability

The data supporting the findings of this study are included within the article. Additional anonymised data are available from the corresponding author upon reasonable request, subject to institutional policies.

## Funding

This work received no specific grant from any funding agency in the public, commercial or not-for-profit sectors.

## **Declaration of competing interest**

The authors declare that they have no known competing financial interests or personal relationships that could have appeared to influence the work reported in this paper.
